# Effects of a DVD-delivered randomized controlled physical activity intervention on functional health in cancer survivors

**DOI:** 10.1186/s12885-021-08608-8

**Published:** 2021-07-29

**Authors:** Elizabeth A. Salerno, Neha P. Gothe, Jason Fanning, Lindsay L. Peterson, Graham A. Colditz, Edward McAuley

**Affiliations:** 1 Division of Public Health Sciences, Department of Surgery, Washington University School of Medicine in St. Louis, St. Louis, USA; 2 Department of Kinesiology & Community Health, University of Illinois at Urbana-Champaign, Champaign, USA; 3 Beckman Institute for Advanced Science and Technology, University of Illinois at Urbana-Champaign, Champaign, USA; 4 Department of Health & Exercise Science, Wake Forest School of Medicine, Winston-Salem, USA; 5 Division of Medical Oncology, Department of Medicine, Washington University School of Medicine in St. Louis, St. Louis, USA

**Keywords:** Cancer, Exercise, Physical function, Physical activity, Survivorship, Telerehabilitation

## Abstract

**Background:**

Supervised physical activity interventions improve functional health during cancer survivorship, but remain costly and inaccessible for many. We previously reported on the benefits of a DVD-delivered physical activity program (*FlexToBa™)* in older adults. This is a secondary analysis of the intervention effects among cancer survivors in the original sample.

**Methods:**

Low active, older adults who self-reported a history of cancer (*N* = 46; *M* time since diagnosis = 10.7 ± 9.4 years) participated in a 6-month, home-based physical activity intervention. Participants were randomized to either the DVD-delivered physical activity program focused on flexibility, toning, and balance (*FlexToBa™*; *n* = 22) or an attentional control condition (*n* = 24). Physical function was assessed by the Short Physical Performance Battery (SPPB) at baseline, end of intervention, and at 12 and 24 months after baseline.

**Results:**

Repeated measures linear mixed models indicated a significant group*time interaction for the SPPB total score (β = − 1.14, *p* = 0.048), driven by improved function from baseline to six months in the *FlexToBa™* group. The intervention group also had improved balance (β = − 0.56, *p* = 0.041) compared with controls. Similar trends emerged for the SPPB total score during follow-up; the group*time interaction from 0 to 12 months approached significance (β = − 0.97, *p* = 0.089) and was significant from 0 to 24 months (β = − 1.84, *p* = 0.012). No significant interactions emerged for other outcomes (*p*s > 0.11).

**Conclusions:**

A DVD-delivered physical activity intervention designed for cancer-free older adults was capable of eliciting and maintaining clinically meaningful functional improvements in a subgroup of cancer survivors, with similar effects to the original full sample. These findings inform the dissemination of evidence-based physical activity programs during survivorship.

**Trial registration:**

ClinicalTrials.govNCT01030419. Registered 11 December 2009

**Supplementary Information:**

The online version contains supplementary material available at 10.1186/s12885-021-08608-8.

## Background

There is a wealth of evidence for the benefits of physical activity during cancer survivorship, ranging from improved functional and psychosocial health to decreased risk of recurrence and mortality [1–4]. However, many of the existing evidence-based physical activity interventions after cancer have been supervised [1], requiring travel to a university or clinical setting. While such interventions are important for understanding physical activity’s efficacy for improving health outcomes, their design inherently limits access for many survivors. Notably, these are individuals who may stand to benefit the most from such programs (e.g., sedentary or rural-dwelling survivors and those who are functionally impaired/disabled), highlighting the need for innovative, scalable physical activity interventions that can be disseminated to the broader population of cancer survivors [5].

As technology advances, these efforts have focused on developing and testing digitally-delivered physical activity interventions during cancer survivorship [6], with small but promising evidence for increased moderate-to-vigorous physical activity (MVPA) [7]. However, many of these trials suffer from several limitations (e.g., single arm, no theoretical framework, short follow-up) that prevent us from understanding how best to implement physical activity in the home for increased physical activity and improved functional health [7, 8]. Before designing new interventions, it is necessary to understand if existing evidence-based, high quality interventions in other populations can successfully increase physical activity and improve functional health in cancer survivors. Such analyses will better inform the design and implementation of cancer-specific trials. Given the accelerated aging phenotype often seen in cancer survivors [9, 10], the aging literature may be a suitable framework for such an analysis.

We previously conducted a DVD-delivered 6-month randomized controlled exercise intervention in older adults across the state of Illinois [11]. The *Flexibility, Toning, and Balance Trial (FlexToBa™)* is grounded in Social Cognitive Theory [12] with a Reach, Effectiveness, Adoption, Implementation, Maintenance (RE-AIM) framework [13], unique elements designed to influence public health [11]. Participants in the intervention arm evidenced significant improvements in functional health, self-esteem, anxiety and depressive symptoms, had reduced sedentary behavior, and maintained physical activity levels up to two years after the intervention compared with the control condition [14–21]. Notably, most of these effects were moderate in size with clinically meaningful implications. *FlexToBa™* has also been successfully delivered to individuals with Multiple Sclerosis (MS), with small to modest effects for improved functional health and quality of life, increased physical activity, and decreased sitting time in the intervention arm [22].

Cancer survivors are now living well into older adulthood, and there are real concerns about healthy aging in this population given the number of functional impairments that persist well into survivorship [23]. It is important to understand if and how physical activity interventions designed for older adults also extend to long-term survivors, or alternatively, require additional cancer-specific tailoring. *FlexToBa™* was designed to be adaptable to different functional abilities, with robust effects both in older adults and clinical populations such as MS, and extensive reach [24]. Thus, *FlexToBa™* is a good candidate intervention for determining the effects of an existing physical activity program in individuals with a history of cancer. To this end, we examined the effects of the intervention on functional health, physical activity, and sedentary behavior in participants from the original sample who self-reported a prior cancer diagnosis. We hypothesized that those in the intervention arm would exhibit similar effects as the full sample, such that survivors would demonstrate improved functional health, maintained physical activity levels, and reduced sedentary behavior at the end of the intervention and after a 12- and 24-month non-contact follow-up period.

## Methods

### Participants, Study Design & Interventions

**Fig. 1:**
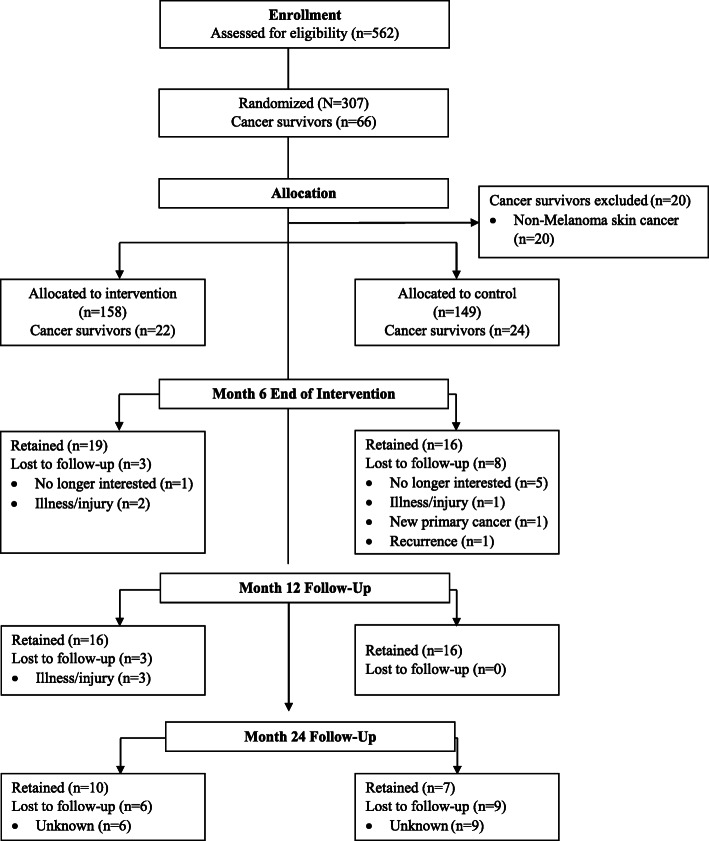
CONSORT for cancer survivors enrolled in *FlexToBa™*. Reasons for exclusions provided for cancer survivors only. Month 24 follow-up added after study initiation, reasons for exclusions not collected

A comprehensive description of the *FlexToBa™* trial, including eligibility criteria, randomization allocation, and intervention details, has been published previously [11]. Briefly, community-dwelling, low active older adults over the age of 65 years were recruited from 83 towns and cities across a 5000 mile^2^ area of central Illinois to participate in a 6-month randomized controlled exercise trial examining the effects of a home-based physical activity intervention on physical activity, functional health, and quality of life. Participants (*N* = 307) were randomized to either the *FlexToBa™* DVD intervention or a healthy aging DVD control condition. The present study represents a secondary analysis of participants who self-reported a history of cancer at baseline of the intervention (*n* = 66). Non-melanoma skin cancers were removed (*n* = 20), resulting in a final sample of 46 cancer survivors for the current analysis. Flow of cancer survivors through the trial is illustrated in Fig. 1.

The intervention arm received the *FlexToBa™* DVD, two resistance bands of varying intensities, a yoga mat, and a *FlexToBa™* handbook. The DVD consisted of six progressive exercise sessions, one for each month of the program, each with 11–12 different exercises focused on flexibility, toning, and balance. The intervention was progressive in nature, both within and across the six months, such that the exercises built on themselves over the course of the intervention. A trained exercise leader led the exercises and was flanked by age-matched individuals demonstrating modified and challenging versions of the exercises. The control condition received a commercially-available DVD by Dr. Andrew Weil, *Healthy Aging*, focused on generic health topics such as sleep, nutrition, exercise, and well-being*.* Both conditions received titrated support telephone calls from intervention staff over the course of the six months; these calls did not continue after formal intervention cessation. Study protocols were reviewed and approved by a university Institutional Review Board (approval #09765) and all participants provided written informed consent prior to participation.

### Measures

All measures were assessed at baseline, end of the intervention (six months), and after two no-contact follow-up periods (e.g., 12 and 24 months after baseline) by blinded research staff.

#### Physical function

The well-validated Short Physical Performance Battery (SPPB) [25] assessed balance, gait speed, and lower extremity strength through timed postural maintenance, timed 4-m walking, and timed chair stands, respectively. Performance scores for individual SPPB tests are provided along with a total summary score aggregating the individual tests. Also measured were upper body strength and endurance using an arm curl test (e.g., number of curls completed in 30 s) and lower body flexibility with a sit and reach test (e.g., +/− inches from toes). Detailed methodology for these tests have been previously reported in the Senior Fitness Test Manual [26].

#### Physical activity

Physical activity was assessed objectively via accelerometry. Participants wore an Actigraph brand accelerometer (Actigraph, Pensacola, FL; Model GT1M or GT3X) on the non-dominant hip for 7 consecutive days and recorded the time spent wearing the accelerometer on a log. Data retained for analysis had ≥10 h of wear-time per day for at least 3 days when scored with an interruption period of 30 min. Data were downloaded as activity counts, representing raw accelerations summed over a specific epoch length (e.g., 60 s) and varied based on frequency and intensity. These data were then processed using older adult-specific cut-points [27]. Sedentary behavior is defined as ≤50 cpm, light physical activity is defined as 51–1040 cpm, and moderate-to-vigorous physical activity (MVPA) is defined as ≥1041 cpm. Total wear time was divided by the number of valid days to represent average daily counts/minute.

#### Functional limitations

The abbreviated function component of the Late-Life Function and Disability Instrument [28] assessed the degree of difficulty experienced by participants with basic and advanced lower and upper extremity function. Participants indicated the difficulty they would have with specific tasks on a 5-point Likert scale ranging from 5 (none) to 1 (cannot do). Subscales were combined to provide a total functional limitations score, such that lower scores indicated fewer difficulties when performing activities of daily living.

#### Intervention components

We further explored adherence to the intervention, assessed quantitatively as number of exercise days per month as reported by participants on home exercise logs. Satisfaction with different aspects of the intervention (e.g., exercise leader, DVD, research team) was measured on a Likert scale from 0 (dissatisfied) to 5 (very satisfied), and participants self-reported up to four benefits of the program (% of sample reporting yes/no to each benefit). We also explored the number of adverse events to assess safety of the intervention after a cancer diagnosis.

### Data analysis

Repeated measures mixed models assessed the effect of the intervention on functional health and physical activity outcomes over: (i) the 6-month intervention and (ii) the entire 24-month follow-up window. Time was calculated as months since baseline of the intervention. Fixed effects included time, group, and their interaction. Random effects were specified at the individual level. All models were estimated using restricted maximum likelihood (REML) method. Significance level for all models was set to 0.05 and all analyses were conducted using intent-to-treat in Stata version 16.1 (StataCorp, College Station, Texas). Across the entire sample, effects of the intervention did not differ in cancer survivors compared to cancer-free participants (*p*s > 0.11), therefore only cancer survivors were retained for analysis.

## Results

Participant characteristics are detailed in Table 1.

**Table 1:** Participant demographics & cancer characteristics at baseline

	Total Sample *N* = 46	FlexToBa *n* = 22	Control *n* = 24
	M (SD) or %	M (SD) or %	M (SD) or %
Age ^†^	72.9 (5.3)	72.1 (5.6)	73.6 (5.0)
Female	80.4%	68.2%	91.7%
White	93.5%	95.5%	91.7%
Body Mass Index	32.0 (6.7)	32.6 (7.3)	31.4 (6.2)
Married	62.2%	61.9%	62.5%
Rural Hometown	32.6%	27.3%	37.5%
Cancer Type			
Breast	37.0%	40.9%	33.3%
Colon	6.5%	9.1%	4.2%
Melanoma	13.0%	6.5%	12.5%
Prostate	6.5%	13.6%	4.2%
Other	37.0%	29.9%	45.8%
Time Since Diagnosis ^†^	10.7 (9.4)	10.4 (8.1)	11.0 (10.6)
Cardiovascular disease Hx*	2.8 (1.7)	2.6 (1.6)	2.9 (1.8)
Musculoskeletal pain Hx*	0.5 (0.7)	0.6 (0.7)	0.4 (0.7)
Pulmonary disease Hx*	0.2 (0.4)	0.1 (0.4)	0.2 (0.4)

M = mean; SD = standard deviation; Hx = history

^†^ Years; *Mean number of reported events

Briefly, individuals were on average 73 years of age [*M*_*age*_ = 72.9 ± 5.3], 80.4% female, and 93.5% White. Average body mass index of participants was 32.0 ± 6.7 and 32.6% resided in rural hometowns. The most commonly reported cancer type was breast (37.0%), followed by melanoma (13.0%). Other cancer types reported were: bladder, cervical, colon, Extramammary Paget’s Disease, endometrial, fallopian tube, kidney, leukemia, lymphoma, prostate, thyroid, tongue, and uterine. Two participants reported multiple cancers and one reported recurrent cancer. Average time since diagnosis was 10.7 ± 9.4 years.

### End of intervention (6-month)

Repeated measures linear mixed models indicated a significant group*time interaction for the SPPB Total Score (β = − 1.14, *p* = 0.048), driven by improved physical performance in the *FlexToBa™* group from baseline to end of intervention, compared with worsened performance in the control group. A similar trend was observed for the SPPB balance score, such that those in the intervention group had better balance at the end of the intervention (β = − 0.56, *p* = 0.041). Trends for physical activity maintenance and decreased sedentary behavior in the intervention group emerged that, while not statistically significant (*p*s > 0.11), demonstrated meaningful effect sizes (light physical activity *d* = − 0.48; MVPA *d* = − 0.22; sedentary behavior *d* = 0.56). We did not observe significant group*time differences for other measures of physical function or functional limitations (*p*s > 0.18). Full results from baseline to end of intervention are detailed in Table 2, and unadjusted mean data at baseline, end of intervention, and follow-up are detailed in Supplemental Table 1.

**Table 2:** Full linear mixed models results from baseline (m0) to end of intervention (m6)

	Model 1			Model 2		
	β (SE)	z	*p*	β (SE)	z	*p*
**SPPB Total Score**						
Group	−0.10 (0.47)	−0.22	0.83	−0.02 (0.47)	0.47	0.96
Time	0.56 (0.40)	1.39	0.16	0.55 (0.40)	1.38	0.17
Group*Time	−1.14 (0.58)	−1.98	0.048*	− 1.13 (0.57)	− 1.98	0.048*
**SPPB Balance Score**						
Group	−0.06 (0.24)	0.24	0.81	0.00 (0.24)	0.01	0.99
Time	0.25 (0.19)	1.29	0.20	0.24 (0.19)	1.28	0.20
Group*Time	−0.56 (0.28)	−2.04	0.04*	−0.56 (0.27)	− 2.06	0.04*
**Arm Curls (per 30 s timed test)**						
Group	−1.08 (1.06)	− 1.02	0.31	− 0.78 (1.03)	− 0.75	0.45
Time	1.77 (0.78)	2.28	0.02	1.75 (0.78)	2.24	0.03
Group*Time	−1.52 (1.12)	−1.35	0.18	−1.50 (1.13)	− 1.33	0.18
**Sit and Reach (inches)**						
Group	−1.16 (1.34)	−0.87	0.38	− 0.89 (1.34)	− 0.67	0.51
Time	−1.48 (0.51)	−2.89	0.004	−1.48 (0.51)	− 2.90	0.004
Group*Time	0.24 (0.74)	0.34	0.76	0.23 (0.74)	0.31	0.76
**LL-FDI Total Score**						
Group	−3.97 (2.12)	− 1.87	0.06	−3.19 (2.00)	− 1.60	0.11
Time	1.33 (1.12)	1.19	0.23	1.29 (1.13)	1.15	0.25
Group*Time	−1.90 (1.63)	−1.17	0.24	−1.86 (1.63)	− 1.14	0.25
**Light PA (average daily minutes)**						
Group	4.07 (19.69)	0.21	0.84	7.63 (19.72)	0.39	0.70
Time	9.42 (13.56)	0.69	0.49	8.42 (13.50)	0.62	0.53
Group*Time	−22.50 (19.59)	−1.15	0.25	−21.22 (19.49)	−1.09	0.28
**MVPA (avg daily min)**						
Group	−8.45 (6.29)	−1.34	0.18	−6.42 (5.91)	−1.09	0.28
Time	−0.86 (5.92)	−0.15	0.88	−2.07 (5.76)	− 0.36	0.72
Group*Time	−4.84 (8.46)	−0.57	0.57	−3.23 (8.22)	−0.39	0.70
**Sedentary Behavior (avg daily min)**						
Group	−2.55 (26.57)	−0.10	0.92	−5.53 (26.82)	−0.21	0.84
Time	−32.40 (24.25)	−1.34	0.18	−30.57 (24.07)	−1.27	0.20
Group*Time	54.99 (34.73)	1.58	0.11	52.81 (34.46)	1.53	0.13

Model 1 = unadjusted; Model 2 = adjusted for age

SE = standard error; SPPB=Short Physical Performance Battery; LL-FDI = functional limitations; PA = physical activity; MVPA = moderate to vigorous physical activity

### Follow-ups (12- and 24-months)

**Fig. 2:**
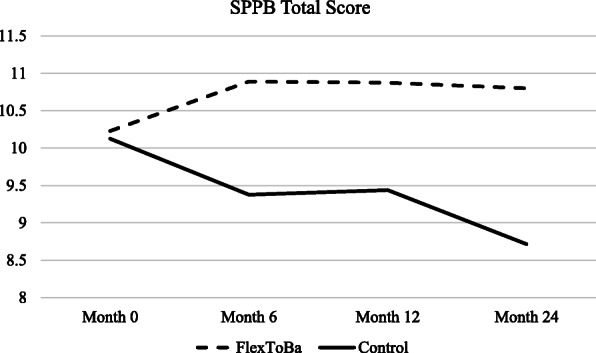
24-month trajectory of SPPB Total Score by group. SPPB=Short Physical Performance Battery. FlexToBa = Flexibility, Toning & Balance Intervention

When examining the effects of the *FlexToBa™* intervention over the follow-up period, similar patterns emerged for the SPPB Total score. The group*time interaction from 0 to 12 months trended towards improved function in the intervention group (β = − 0.97, *p* = 0.089) and was significant from 0 to 24 months (β = − 1.84, *p* = 0.012). Figure 2 depicts the 24-month trajectory. No further group differences were observed over the follow-up period for functional health (*p*s > 0.11).

### Intervention components

No adverse events occurred in the sample of cancer survivors. Adherence in the exercise group gradually declined over the course of the intervention. Average exercise days for each month were as follows: month 1 = 12.0 ± 3.0; month 2 = 8.7 ± 4.4; month 3 = 8.1 ± 4.5; month 4 = 7.0 ± 5.2; month 5 = 5.6 ± 5.3 and; month 6 = 5.5 ± 5.5 days. Overall, participants in the *FlexToBa™* group enjoyed the intervention. On a scale of 0 (dissatisfied) to 5 (very satisfied), average ratings of the exercise program, exercise leader, quality of the DVD, progression of the exercises, modifications available, testing appointments, support calls, and the *FlexToBa™* research team were all above 4.5. The most common self-reported benefits of the program included physical health (43.5%), mental health (13.0%), and behavior regulation (10.9%).

## Discussion

Physical activity during cancer survivorship is widely encouraged but remains poorly implemented, particularly in both clinical and community settings [6, 27]. Our findings suggest that a DVD-delivered intervention originally designed for older adults provided significant improvements in functional health after cancer. Importantly, these effects were maintained over a year after the end of the intervention. These findings are consistent with the body of evidence documenting functional benefits with increased physical activity during cancer survivorship [1, 2], and support the broad dissemination of general physical activity programs to long-term cancer survivors. However, our findings suggest that intervention tailoring is warranted for increasing physical activity levels and further optimizing health benefits during cancer survivorship.

Our finding of the positive effects of physical activity on functional health is consistent with the exercise oncology literature [8]. A consensus statement from an international multidisciplinary roundtable of experts documented there is strong evidence that physical activity improves physical functioning in cancer survivors [1]. However, more robust effects of exercise have been documented in supervised interventions [1], which may be due to the presence of other participants, trained personnel and social support, as well as home-based interventions’ heavy reliance on self-reported measures with limited to no follow-up [7, 8]. This study documents the efficacy of a home-based exercise program for improving functional health, as assessed by the SPPB, after cancer with over a year of follow-up. Importantly, effects of the intervention did not differ between older adults and those with a history of cancer. The mean SPPB score in the intervention cancer group was a full point higher than the control cancer group at the end of the intervention, representing a clinically meaningful difference [29]. No adverse events occurred in this subsample of cancer survivors, highlighting the safety of such a program for this specific sample of survivors. Of course, it should be noted that this sample was comprised of long-term survivors (mean time since diagnosis = 10.7 years) who were deemed healthy enough by their personal physicians to participate. Only 8 participants were less than two years from their cancer diagnoses, which limited our ability to determine if the intervention effects may have been modified by acute tumor- or treatment-related factors. While future work would do well to replicate these findings in cancer survivors who are in the early stage of their disease and/or closer to treatment, our findings have important public health and safety implications for the dissemination of general, home-based physical activity programs for long-term cancer survivors.

The successful DVD delivery of this intervention to cancer survivors has important connotations for extending our reach beyond university and clinical settings to a broader range of survivors, including those who have limited healthcare access, a functional disability, and/or reside in rural environments. Recent research suggests that it may be a feasible and acceptable dissemination method in this population [29–31]. Indeed, several reviews have noted that most cancer survivors prefer to exercise at home with flexible programs that can be adapted to fit an individual’s schedule; however, most survivors also prefer tailoring to cancer-specific content [7, 32]. Roberts and colleagues [7] further highlighted that while digitally-delivered behavior change interventions in cancer survivors can successfully increase physical activity levels across different platforms (e.g., website, mobile phone), the risk of bias and heterogeneity in these trials is high [33]. The present study included accelerometry and over two years of follow-up, but only 46 of the 307 participants in the original sample reported a history of cancer. There is a clear need for larger randomized controlled trials with objective measures of physical activity, long-term follow-up, and standardized measures of health [34]. Ideally, these trials would be designed with implementation in mind and include: i) content specific to the cancer experience and symptomology for safety, comfort, and preference; ii) quantitative and qualitative feedback for further optimization and; iii) objective, generalizable measures of both physical activity and health outcomes, including those that are cancer specific, with long follow-up periods.

Despite the ease of delivering this intervention via DVD over a six-month period, it’s unclear how this medium would be accepted over the next several years with new emerging technologies and growing geographic disparities after cancer [35]. This content may be readily transferrable to newer technologies such as smart phones or mobile applications (or even remain in DVD format); however, future work should seek to understand survivors’ preferences about delivery mode and access to technology for home-based physical activity programs before making adaptations. While certain physical activity preferences have generally been well-studied in cancer survivors (e.g., physical activity modality, location, content) [36], less is known about the extent to which rural-dwelling survivors are able to access specific technologies required for digitally-delivered interventions (e.g., internet access, electronics ownership). A recent systematic review and RE-AIM evaluation of rural physical activity interventions indeed highlighted limited intervention effects and generalizability, likely due to poor reporting across trials [37]. While *FlexToBa™* was largely well-attended and received based on anonymized feedback, we were unable to determine how feedback varied between cancer survivors and the full sample, or in those rural-dwelling individuals with reduced access to technological support. Maintaining high levels of adherence in an unsupervised, rural environment is another important safety and efficacy concern as future efforts strive to deliver accessible interventions via technology. While adherence in this sample gradually declined over the course of the intervention, potentially due to tapered contact from research staff, participants were still engaging with the DVD more than once a week. More contemporary technologies would allow for real time, objective monitoring of usage patterns, which may be leveraged to promote adherence and ensure safety. If our goal is to bring physical activity benefits to all cancer survivors, more work is required to understand how to maximize and optimize our reach across cancer populations to promote equitable access.

Of further interest is the lack of significant intervention effects on objective physical activity and sedentary behavior in this sample of cancer survivors. *FlexToBa™* placed a large emphasis on strengthening and balance exercises, and the current sample of cancer survivors was small; therefore, it is not surprising that we were unable to detect significant changes in aerobic physical activity. Our effect sizes do indicate, however, that programs like *FlexToBa™* may still be meaningful and important for physical activity profiles. Strengthening and balance are important in the context of fall prevention after treatment for breast cancer [38], especially in older survivors. To optimize health and activity improvements based on recommendations by the Department of Health and Human Services [39], future interventions would benefit from including a walking component. Most survivors are physically capable of walking and have identified it as a preferred modality for physical activity programs [36], making it a feasible and clearly desirable addition to technologically-delivered home-based interventions like *FlexToBa™.*

These findings should be interpreted in the context of their strengths and limitations. While the current sample included fewer than 50 individuals with a history of cancer, *FlexToBa™* is a large, randomized controlled exercise trial with a theoretical framework, objective measures of physical activity and health, and long follow-up periods: a solid foundation for future trials to build upon. Participants self-reported their adherence to the intervention, which may introduce social desirability bias, and their cancer diagnoses; no information on treatment regimen or cancer stage was available. These effects may be different in survivors of different stages, times since diagnosis, and/or treatment regimens. Nonetheless it is encouraging that a physical activity intervention originally designed for older adults was capable of improving functional health in long-term cancer survivors. The current sample was predominantly female, which is unsurprising given that there are proportionately more women aged 65 years and older than men; however, we were unable to recruit a larger percentage of minority participants despite our increased recruitment efforts in areas with higher numbers of minority adults. These findings should be replicated in larger samples of more racially and geographically diverse cancer populations to better understand how physical activity can be successfully implemented into different communities. Additional tailoring may be warranted to reach minority populations to ensure that all survivors can receive physical activity benefits.

## Conclusions

We observed similar benefits in the effects of a DVD-delivered physical activity intervention on physical function between older adults and those with a history of cancer. In cancer survivors specifically, those in the intervention arm demonstrated significant improvements in functional performance compared to those in the control condition. These findings point to the need for risk stratification during cancer survivorship so that low-risk survivors can be guided to general physical activity programs such as *FlexToBa™* for improved and maintained health during long-term survivorship. Further cancer-specific adaptations may be necessary to maximize health benefits of these home-based programs, and it remains of paramount importance to increase access and adherence to physical activity programs for survivors in low-resource settings for improved health and longevity.

## Supplementary Information

**Additional file 1.**

## Data Availability

The datasets used and/or analyzed during the current study will be made available upon reasonable request from the corresponding author; the data are not publicly available due to privacy or ethical restrictions.

## Abbreviations

FlexToBa™: Flexibility, toning, and balance trial
SPPB: Short Physical Performance Battery
MVPA: moderate to vigorous physical activity
RE-AIM: Reach, Effectiveness, Adoption, Implementation, Maintenance
MS: multiple sclerosis
REML: restricted maximum likelihood estimation
M: mean
SD: standard deviation
SE: standard error
LL-FDI: functional limitations
