# Supplementary material for: Effects of a DVD-delivered randomized controlled physical activity intervention on functional health in cancer survivors
Source: BMC Cancer. 2021 Jul 29;21:870. doi: 10.1186/s12885-021-08608-8 (PMC8323277; doi:10.1186/s12885-021-08608-8)
Supplement: Supplementary file 1 — Additional file 1. [file 12885_2021_8608_MOESM1_ESM.docx]

*Supplemental Table 1. Unadjusted mean data at baseline, end of intervention, and follow-up by group*

|  | **Total Sample**  **M (SD)** | **FlexToBa**  **M (SD)** | **Control**  **M (SD)** |
| --- | --- | --- | --- |
| **SPPB Total Score** | |  |  |
| M0 | 10.2 (1.3) | 10.2 (1.3) | 10.1 (1.3) |
| M6 | 10.2 (2.0) | 10.9 (1.0) | 9.4 (2.5) |
| M12 | 10.2 (1.9) | 10.9 (1.2) | 9.4 (2.3) |
| **SPPB Balance Score** | |  |  |
| M0 | 3.7 (0.7) | 3.7 (0.8) | 3.6 (0.7) |
| M6 | 3.6 (0.9) | 4.0 (0.0) | 3.3 (1.3) |
| M12 | 3.6 (0.8) | 3.6 (0.6) | 3.5 (1.0) |
| **Arm Curls (per 30 second timed test)** | | |  |
| M0 | 13.4 (3.5) | 14.0 (3.1) | 12.9 (3.8) |
| M6 | 14.5 (4.0) | 15.7 (3.3) | 13.1 (4.3) |
| M12 | 14.2 (3.4) | 15.1 (3.4) | 13.3 (3.3) |
| **Sit and Reach (inches^Ŧ^)** | |  |  |
| M0 | -0.13 (4.2) | 0.5 (3.5) | -0.7 (4.8) |
| M6 | -1.8 (5.1) | -0.8 (4.9) | -2.9 (5.3) |
| M12 | -0.4 (4.0) | 0.4 (3.0) | -1.3 (4.7) |
| **LL-FDI Total Score** | |  |  |
| M0 | 61.1 (7.0) | 63.1 (8.1) | 59.2 (5.4) |
| M6 | 62.6 (7.7) | 65.1 (7.6) | 59.8 (7.1) |
| M12 | 62.6 (7.5) | 66.1 (5.4) | 59.2 (7.8) |
| **Light PA (average daily minutes)** | |  |  |
| M0 | 271.1 (69.5) | 268.8 (80.4) | 273.4 (59.1) |
| M6 | 271.3 (62.6) | 285.5 (63.3) | 256.1 (60.0) |
| M12 | 268.7 (54.6) | 274.5 (59.3) | 262.6 (50.6) |
| **MVPA (avg daily min)** | |  |  |
| M0 | 28.9 (23.5) | 33.3 (24.8) | 24.7 (21.9) |
| M6 | 26.4 (18.6) | 33.1 (19.2) | 19.4 (15.6) |
| M12 | 26.7 (16.3) | 35.0 (16.8) | 17.7 (10.2) |
| **Sedentary Behavior (avg daily min)** | | |  |
| M0 | 551.9 (102.1) | 553.5 (133.0) | 550.5 (63.2) |
| M6 | 544.1 (68.9) | 517.5 (63.0) | 572.5 (65.1) |
| M12 | 541.0 (56.1) | 536.0 (46.9) | 546.3 (65.9) |

^Ŧ^ Negative values indicate more flexibility (greater distance reached beyond toes)

M0=baseline; M6=end of intervention; M12=follow-up; SPPB=Short Physical Performance Battery; LL-FDI=functional limitations; PA=physical activity; MVPA=moderate to vigorous physical activity
